# ICPD-A New Peak Detection Algorithm for LC/MS

**DOI:** 10.1186/1471-2164-11-S3-S8

**Published:** 2010-12-01

**Authors:** Jianqiu Zhang, William Haskins

**Affiliations:** 1 Department of Electrical Engineering, University of Texas at San Antonio, San Antonio, Texas, USA; 2 Department of Biology, University of Texas at San Antonio, San Antonio, Texas, USA

## Abstract

**Background:**

The identification and quantification of proteins using label-free Liquid Chromatography/Mass Spectrometry (LC/MS) play crucial roles in biological and biomedical research. Increasing evidence has shown that biomarkers are often low abundance proteins. However, LC/MS systems are subject to considerable noise and sample variability, whose statistical characteristics are still elusive, making computational identification of low abundance proteins extremely challenging. As a result, the inability of identifying low abundance proteins in a proteomic study is the main bottleneck in protein biomarker discovery.

**Results:**

In this paper, we propose a new peak detection method called Information Combining Peak Detection (ICPD ) for high resolution LC/MS. In LC/MS, peptides elute during a certain time period and as a result, peptide isotope patterns are registered in multiple MS scans. The key feature of the new algorithm is that the observed isotope patterns registered in multiple scans are combined together for estimating the likelihood of the peptide existence. An isotope pattern matching score based on the likelihood probability is provided and utilized for peak detection.

**Conclusions:**

The performance of the new algorithm is evaluated based on protein standards with 48 known proteins. The evaluation shows better peak detection accuracy for low abundance proteins than other LC/MS peak detection methods.

## Background

The identification and quantification of proteins in biological samples play crucial roles in biological and biomedical research [1-3]. For example, in biomarker discovery studies, the aim is to elucidate a set of proteins that can be used to reliably differentiate diseased and normal samples. Accurate protein identification and quantification are required to achieve this goal.

Many researchers have pointed out that biologically meaningful proteins are often of low abundance [2], therefore, the percentage of low abundance proteins identified determines the likelihood of success in biomarker discovery studies. Recent improvements in sample preparation and instrumentation directly address this problem. In contrast, the current data processing algorithms for low abundance proteins have been a neglected component of protein biomarker discovery. This reality has underscored the need for the improvement of peak detection algorithms for low abundance proteins.

In this paper, we focus on label-free Liquid Chromatography/Mass Spectrometry (LC/MS) for protein/peptide identification and quantification. Although currently, most of the quantitative proteomics approaches by mass spectrometry utilize isotopic labels such that samples from difference class/disease states can be analyzed in one LC/MS run. However, the capacity of the LC column must be shared by the number of isotopically- or isobarically-labeled samples to be compared, which severely limits the sensitivity for observing the low abundance proteins; consequently, the use of label-free quantification methods are preferred.

It is possible to employ LC/MS/MS, or LC-tandem mass spectrometry for biomarker discovery. However in [4], it is demonstrated that while 80 to 98% of the most abundant proteins can be identified in a triplicate LC/LC/MS/MS experiment, only 10 to 25% of lower abundance proteins, which are the vast majority, were identified. The limitations of LC/MS/MS also preclude the replicate analysis required to gather statistical information from a large number of samples and often only the most abundant ’housekeeping proteins’ are observed. Thus, while critical to identify low abundance proteins from complex biological mixtures, LC/MS/MS and LC/LC/MS/MS are less ideal than label-free LC/MS for biomarker discovery applications

### Challenges in the detection of low abundance proteins by capillary LC/MS

The high resolution separation of peptides by LC improves the sequence coverage of low abundance proteins by MS. However, such approach also complicates the subsequent detection of low abundance proteins from noise by a number of factors: 1) Information of peptides is dispersed to multiple locations. A peptide species will register peaks in a series of MS scans within its elution period. 2) Within an MS scan, a peptide species could register several peaks in different charge states and isotope positions. 2) LC/MS systems are subject to considerable noise and variability, which have not been characterized completely. It is critical to perform computational peak picking to tease apart noise and peptide peaks. Peak detection effects biomarker discovery the most because error made at this stage will propagate to subsequent processing steps in biomarker discovery.

Most peak detection algorithms proposed to date focus on the detection of high abundance peptides and cannot meet the requirement of low abundance peptide peak detection mainly due to the following two reasons: 1) Current algorithms do not consider all possible signals generated by a peptide spices for peak detection. Most of software packages including Peplist [5], VIPER [6], SuperHirn [7], OpenMS [8], and msInspect [9] perform peak detection in one charge state and one MS scan. Then, peaks detected in single scans are linked together to form LC peaks. 2) There lacks accurate signal and noise models. For instance, MapQuand [10] assumes a Gaussian model for the elution peak and treats LC peaks that do not conform to the model as noise. However, a lot of real LC peaks, especially low abundance ones, do not conform to the Gaussian shape. Also, the noise models are highly instrumental dependent. For example, in [11], MS noise for Ion-trap instruments is modeled as Poisson but in [12], the LC dimension noise is modeled to have a variance that grows quadratically with signal intensity. The distribution of noise is also instrumental dependent and generally unknown. The optimal peak picking algorithm has to be adapted to different noise models. However, current algorithms generally assume one noise model (Gaussian) and use a fixed model for different instruments and intensity ranges. Deficiencies in these two aspects clearly explain why current peak picking methods cannot utilize 2D LC/MS data effectively for low abundance peptide detection and quantification [13]. There exists a huge potential for performance improvement of peak identification algorithms. In this paper, we propose a novel peptide peak identification algorithm-Information Combining Peak Detection (ICPD) to address this problem.

### Signals generated by a peptide

A peptide species with molecular weight *m* may generate a group of related peaks in an LC/MS dataset. First of all, when a peptide species enters the mass spectrometer, different number of charges will be attached during the ionization process, which results in different charge states. Apart from peptide charge state dispersion, each peptide species would register as a series of isotope peaks in MS. Given the total count of a peptide species, the percentage of the peptide with ’*iso*’ carbon isotopes is governed by the Poisson distribution [14,15], and it is referred as an isotope pattern, ratio or distribution *f*(*iso*). It shall be noted that other chemical elements such as Oxygen also contribute to the isotope pattern. However, C^13^ is the dominating factor. There exists various methods [16-18] addressing the calculation of isotope patten. One of the most popular is based on “averagine”, an averaged molecular formula for peptides [16]. Using the “averagine” molecular formula, one can estimate the number of Carbons/Oxygens etc. contained in a peptide sequence given the total molecular mass, which in turn will allow for the calculation of an estimated isotope pattern. The details of theoretical isotope pattern calculation can be found in [16]. The presence of isotope patterns predicted by the “averagine” is an important evidence on the existence of peptides since non-peptides that do not have similar chemical composition as the “averagine” will not register similar isotope patterns as that of peptides.

Isotope and charge state dispersion result in a phenomenon where multiple peaks will be registered for one peptide species in MS spectrums at different m/z locations. Also, at these m/z locations, similar chromatographic peaks will occur in their elution time profiles. These facts enormously complicates the accurate identification of peptide identity.

## Results and discussion

### Performance evaluation method

A fair way for evaluating the performance of peak picking methods is to compare their ROC curves, which plot the false positive rate vs. true positive rate as the threshold on peak picking criteria varies. Suppose the list of peak candidates produced by an LC/MS processing algorithm is *Outlist* with *N* peaks. Each item in the list is annotated by its mass and peak picking parameters such as the isotope pattern matching score. Then a threshold can be applied to one of the parameters such as isotope matching score. Peak candidates that pass the threshold will be treated as detected peaks. Detected peaks consist of True Positives (TP) and False Positives (FP), which can be determined by comparing the detected peaks with the set of *P* “true peptides” obtained using some peak identification methods such as LC/MS/MS. The rest of peaks that do not pass the threshold can also be partitioned to False Negatives (FN) and True negatives (TN) by comparing them to the true peptide list. The true positive rate is estimated as *TP*/(*TP* + *FN*), which indicates the probability of detecting a true peak. The false positive rate is estimated as *FP*/(*TN* + *FP*), which indicates the probability of false peaks being detected as peptide peaks. As we adjust the thresholds, more or less peaks will be detected and the false and true detection rates also change. Tracing these changes will result in the ROC curve. Note that the true positive rate is equivalent to the sensitivity of the algorithm and 1 — *FP* is the specificity of the algorithm on peak detection. The performance of a peak picking algorithm is better when its true detection rate is higher at a given false detection rate, which is reflected as larger area under the curve (AUC). Besides the ROC curve, it is also useful to plot the precision-recall curve [19]. Precision equals True positive rate, and recall rate can be calculated as *recallrate* = *TP/TP* + *FP*. The precision-recall curve is useful when large false positives exists.

### Test data preparation

To verify the performance of the proposed algorithm, it is important to obtain a test dataset. In research such as ([20]), a mixture of 16 known peptides is used for performance evaluation. However, it is hard to draw statistically significant conclusions on the performance of peak detection algorithms based on such a small number of peptides. It is also impractical to manually mix large number of know peptides for testing. Instead, we elect to use the LC/MS dataset generated using the Proteomics Dynamic Range Standard Set (UPS2) from *SIGMA - ALDRICH^TM^*. The UPS2 set is comprised of one vial of Proteomics Dynamic Range Standard and one vial (20 mg) of Proteomics Grade Trypsin. The Proteomics Dynamic Range Standard is produced from a mixture of 48 individual human source or human sequence recombinant proteins, each of which has been selected to limit heterogeneous post-translational modifications (PTMs). The protein standard has a dynamic range of 6 orders of magnitude, ranging from 0.5 fmoles to 50,000 fmoles. The total protein content in each vial is 10.6 mg. Each protein has been quantified by amino acid analysis (AAA) prior to formulation.

Although protein content in UPS2 is known, however, peptide components after trypsin digestion is unknown. Theoretical prediction can provide the list of all possible peptide species when allowing multiple miscleavages of the protein by enzyme. Some entries in the theoretically predicted list have very low probability of occurrence and do not register any signal on the instrument. Thus this theoretically predicted list can not be treated as the “true peptide list”. To establish the “ground truth”, we injected UPS1(*SIGMA - ALDRICH^TM^*) sample which contains the same set of 48 proteins as in UPS2, but with higher protein concentration for LC/MS/MS analysis.

The UPS1 sample was analyzed using an FTMS mass spectrometer (LTQ-Orbitrap-XL, ThermoFisher, San Jose, CA). both LC/MS and LC/MS/MS scans were collected in this experiment. LC/MS/MS of UPS1 data was searched with the MASCOT protein identification algorithm. Mascot returns a list of probable proteins based on MS/MS spectrums. The Mascot search result is then compared to the original protein list. Out of the 283 probable proteins returned by the Mascot search results, 46 out of the original 48 proteins contained in the sample are present. We treat the set of observed peptides in LC/MS/MS scans associated with these 46 proteins as the set of “true peptides” denoted as *L_peptide_* with size *P* that is contained in the trypsin digested sample. Note that this list of 800 peptides can not be the complete set of peptide contained in the UPS2 sample due to a separate trypsin digestion process, however, it is a very close approximation which can be used to compare the performance of various peak picking algorithms.

Subsequently, we process the LC/MS dataset of UPS2 by the ICPD algorithm we proposed that combines information in multiple MS scans and multiple charge states. The algorithm produces a candidate peptide peak list. Each entry in the list is annotated by mass value, elution time period and a score (4) that indicates how likely the peak candidate is a peptide peak.

### Effect of information combining

**Figure 1:**
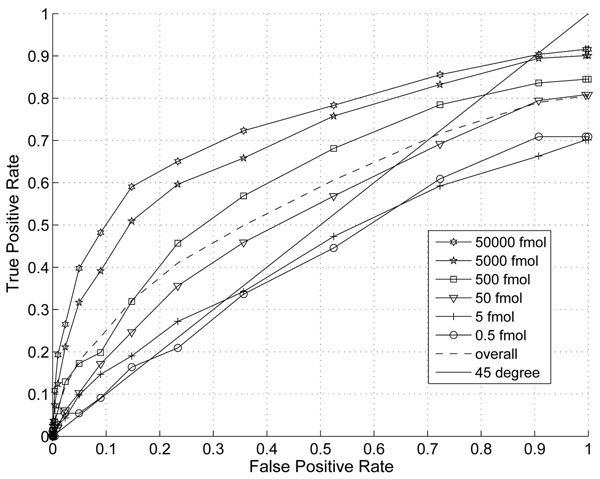
ROC curve with thresholds on proposed score (p=3)

**Figure 2:**
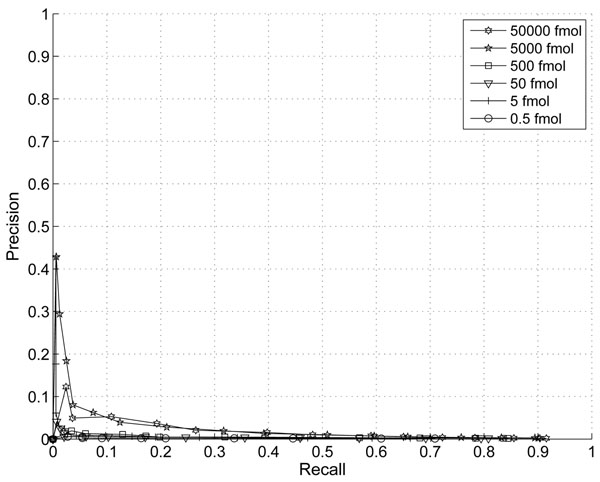
Precision-Recall Curve with thresholds on proposed score (p=3).

**Figure 3:**
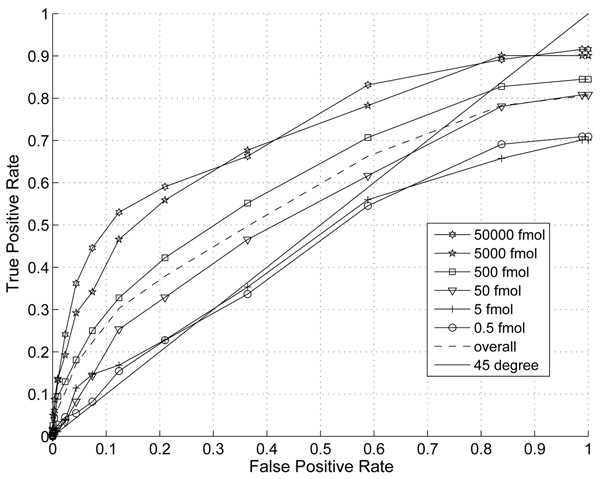
ROC curve with thresholds on proposed score without information combining (p=3).

**Figure 4:**
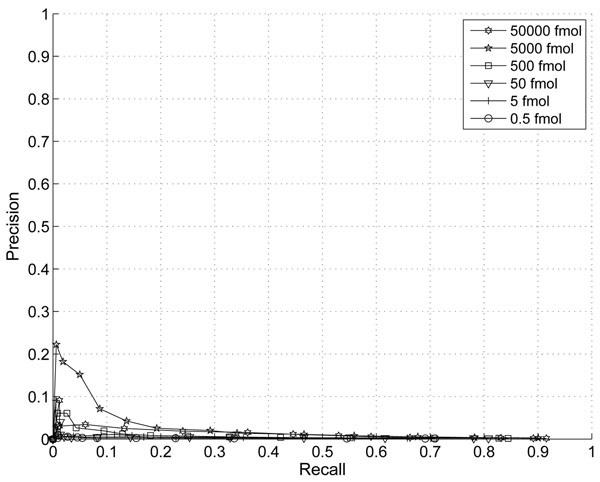
Precision-Recall curve with thresholds on proposed score without information combining (p=3).

In this section, we demonstrate the effect of the information combining. In Fig. 1 and Fig. 2, we plotted the ROC and Precision-Recall curves of the proposed algorithm when different thresholds are applied. The noise variance is assumed to scale with the third power of peptide abundance (p=3). In Fig. 3 and Fig.4, we plotted the ROC and Precision-Recall curves based on a single MS scan with the highest peak intensity. In this experiment, the isotope pattern is estimated based on a single scan without combining information from multiple scans. Then the matching score is calculated similarly as in the case with information combining and the total peptide abundance is assumed to be the highest peak intensity of the peptide candidate.

In the presence of large false positives, the percentage of correct detection is a critical. A precision-recall curve reflects this critical aspects well. While the improvement on ROC curve is not obvious, a clear improvement on precision is shown especially for peptides with abundance of 5000fmol and 5fmol at the same recall rate. The highest precision is at around 43% with our proposed score and only 23% for the single scan case. This clearly shows that the combining of multiple scans improves the performance for lower abundance peptides.

### Effects of noise variance assumption

**Figure 5:**
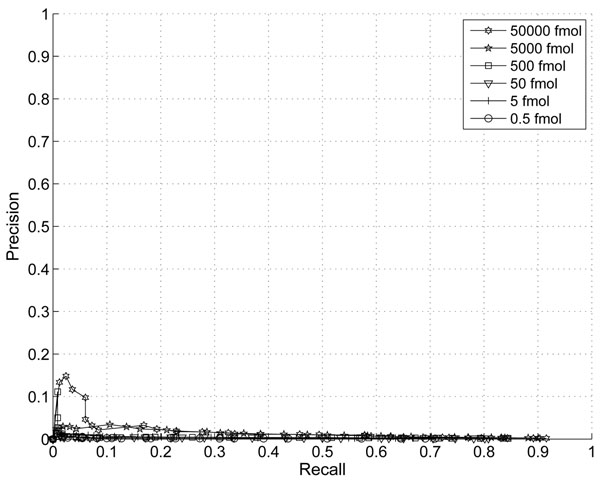
Precision-Recall Curve with thresholds on proposed score p=1

**Figure 6:**
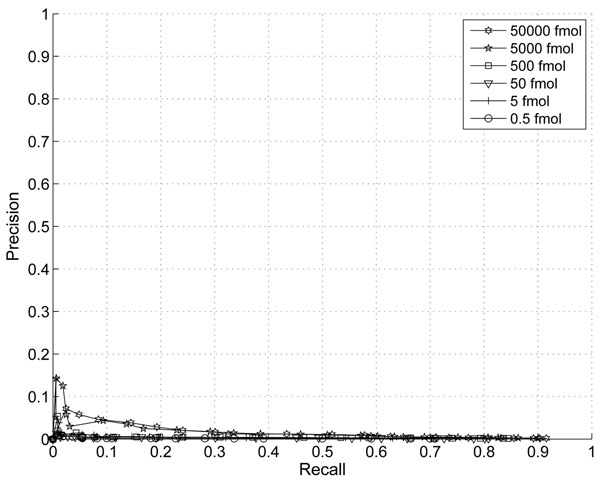
Precision-Recall Curve with thresholds on proposed score p=2

We experimented with different choice of noise variance assumptions. If the poisson model predicted in [11] prevails than the noise variance should scales with the total abundance and the parameter *p* =1. In this case the SNR should increase linearly with total abundance (or volume). If we choose the same model as adopted in algorithms such as VIPER, then a quadratic growth of noise power is implied and *p* = 2. In this case the SNR is assumed to be linear. We also experimented with *p* = 3 which implies that SNR actually gets worse as the peak intensity grows. The precision curves with *p* = 1, *p* = 2, and *p* = 3 are show in the Fig. 5, 6, and 2.

These curves indicate that *p* = 3 gives the best peak picking performance overall. However, it is interesting to notice that with *p* = 1, peak detection on peptides with the highest abundance level provides the best performance. This suggests that the poisson model in [11] is more accurate in high intensity regions and it is necessary to adapt peak detection strategies on different peak intensity regions.

### Comparing proposed score with other peak picking criteria

**Figure 7:**
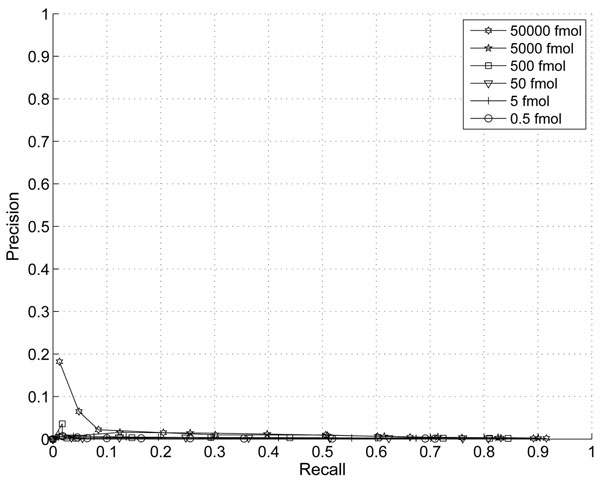
Precision-Recall curve with threshold on Peak Volume based on candidate peaklist reported by ICPD algorithm

Peak detection can be based on various parameters such as peak volume, maximum peak intensity, and LC scan length. These parameters have all been used in the past for peak picking. How does the proposed score compare with other peak picking criteria? Among many criteria reported using our peak list, peak volume reports the best performance. We plotted the Precision-Recall curve based on peak volume in Fig. 7. Comparing Fig. 2 and Fig. 7, it is clear that the proposed score improves the precision of peak detection by more than one fold for peaks with lower abundance although the precision for higher abundance peaks is lowered. This shows that the proposed score improves the precision for lower abundance peaks greatly. For higher abundance peaks, a simple volume peak picking algorithm should be sufficient.

### Comparing to other algorithms

Note that direct comparison to software packages is generally not possible since software packages like msInspect and MZmine offer many options for peak detection. Rather, only peak picking criteria can be compared. Here, we present peak picking results using the software package msInspect for corroborating our results. msInspect is also used for comparison in recent works of [20]. msInspect produces a peptide peak list annotated by various peak parameters such as mass value, elution time period, peak intensity, total peak intensity and KL distance (a type of matching score). It also allows the user to set up thresholds in peak intensity, SNR and other parameters. By setting all thresholds to their most admissible values, msInspect can report all probable peptide peaks which allow us to plot ROC curves for comparison. The peak detection algorithms in some other software packages can find similar parts in msInspect. For example, VIPER employs isotope matching in each scans whose performance is found to be very similar to the result produced by msInspect when using KL distance as the peak picking criteria. Algorithms that employ LC peak shape matching like MZMine and MapQuand generally perform a lot worse than msInspect when processing our LC/MS data set. It is observed that significant amount of LC peaks do not conform to Gaussian or extended Gaussian shape as assumed in MZMine and MapQuand. The algorithm described in [20] is not compared since the algorithm is focused on separating overlapping LC and MS peaks which is a common problem to low resolution data sets. Otherwise, the algorithm is very similar to msInspect.

**Figure 8:**
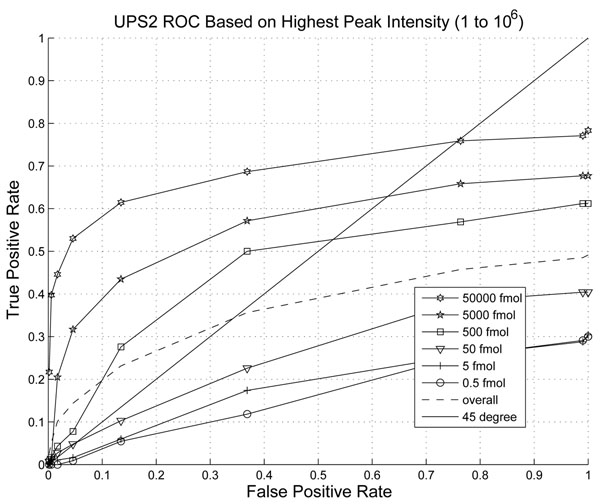
ROC of peak detection on Intensity msInspect.

In Fig. 8, we plotted the ROC curve of the peak picking algorithm by msInspect. We applied thresholds to various parameters and found that peak intensity provides the best ROC performance which is plotted in Fig. 8. The ROCs of the lowest three abundance peptides are lower than the 45 degree line with significantly smaller area under the curve than that of the proposed ICPD algorithm. The maximum detection rate of the low abundance peptides is around 0.3-0.5. We can see a significant improvement in sensitivity and specificity for the detection of peptides with lower abundance provided by our ICPD algorithm. The precision-recall curve reflects the same performance trend and is omitted here.

### Conclusions

In this paper, we proposed a new peak detection algorithm-ICPD for high resolution LC/MS data by combining information. We demonstrated significant improvement in precision for lower abundance peptides over other algorithms. We demonstrated that by assuming a noise model with variance that scales with the third power of peptide abundance, the peak detection performance for low abundance peptides can be improved. This shows that peak picking algorithms have to be adapted to the noise characteristics of instruments.

## Methods

### The information combining peak detection (ICPD) algorithm for LC/MS

In this section, we describe the proposed ICPD algorithm. Isotope pattern matching is widely used for peak detection in MS because non-peptide contaminants do not possess similar isotope patterns as that of peptides. While it is possible to differentiate peptide peaks from noise peaks based on other parameters, such as peak intensity, they are generally not as effective for low abundance peptide identification. Isotope pattern matching has been employed as the core method in many existing LC/MS processing packages ( [6,9]). However, isotope pattern estimation is primarily performed on a scan by scan basis at one charge state at a time. Since LC/MS is corrupted considerably by noise, the estimation of isotope pattern is often inaccurate which greatly reduces the specificity and sensitivity of peak detection.

In the following, we first propose a method to estimate isotope pattern through combining all relevant information in all MS scans and then discuss the the ICPD algorithm which performs peak detection based on the estimated isotope pattern.

#### Isotope pattern estimation based on information combining

Suppose that through some preprocessing steps, which we will describe in detail in the next subsection, a peptide candidate peak list is generated. This candidate list includes all probable peptides peaks. Each entry in the candidate list is a peptide candidate annotated with its mass value and chromatographic peak duration. Suppose that the chromatographic peaks of each peptide candidate have been extracted for all charge states and isotope positions from the LC/MS dataset. For a peptide candidate *i* in the list, we consider the following model for abundance of the chromatographic peak at a particular charge state *cs* and *isotope* position *iso*:

*P_i_*(*t_r_, cs, iso*) = *A_i_ · C_i_*(*tr*) *· f* (*cs*) *· f* (*iso*), (1)

where *A_i_* stands for the total abundance of the *i*th peptide candidate which is also noted as the total volume of the peak, *C_i_*(*t_r_*) stands for the chromatography eluting distribution function of the *i*th peptide candidate at retention time *t_r_*, it describes the fraction of peptide *i* that elutes out during the *t_r_*th retention time period. *C_i_*(*t_r_*) sums to one. *cs* stands for charge state, *iso* stands for the number of Carbon *C*^13^s in the peptide, *f*(*cs*) stands for the charge state distribution function, and *f*(*iso*) stands for the isotope distribution. Both *f*(*cs*) and *f*(*iso*) are distribution functions that sum to one. This model describes how does a peptide elute out according to an elution distribution function and subsequently get dispersed to different charge states according to *f*(*cs*) and isotope positions according to *f*(*iso*). The observed chromatographic peak of the peptide can be expressed as

*y_i_*(*t_r_, cs, iso*) = *p_i_*(*t_r_, cs, iso*) + *n*(*MZ*(*M_i_, cs, iso*)*, t_r_*), (2)

where *MZ*(*M_i_, cs, iso*) stands for the m/z value of the peptide with mass *M_i_* at charge state *cs* and isotope position *iso*. It is calculated as: *MZ* (*cs, iso, M_i_*) = (*M_i_* + *cs · w_p_* + *iso · w_n_*)/*cs*, where *w_p_* stands for the weight of a proton that carries one positive charge, and *w_n_* stands for the weight of a neutron which causes weight shift in an isotope. *n*(*MZ*(·),*t_r_*) stands for the noise at *MZ*(·), and at the elution time *t_r_*. Various sources could attribute to the noise term *n*(*MZ*(*M_i_, cs, iso*), *t_r_*) whose characteristics are not entirely understood and are highly dependent on the instrument [11,12]. Although in studies like [12], the noise variance is studied, the noise distribution is still unknown. Unless an accurate model can be constructed for *n*(*MZ*(*M_i_,cs,iso*), *t_r_*) for the specific instrument studied, effective combining of *y_i_*(·)s can not be performed.

On the other hand, if we sum *y_i_*(*tr, cs, iso*) with respect to *cs* and *C _i_*(*t_r_*), the resulted signal becomes(3)

where  is a noise corrupted copy of *f*(*iso*) with a multiplication coefficient. The noise term  is the summed noise term. Since *n′* is the summation of many noise terms *n*(*MZ*(*M_i_, cs, iso*), *t_r_*) at different m/z locations and MS scans, it can be assumed to be Gaussian according to the central limit theorem. Here, it is characterized as a zero mean Gaussian noise with variance . Given this model,  becomes a Gaussian variable with mean *A_i_f*(*iso*) and variance , where *f*(*iso*) is the theoretically predicted isotope pattern based on “averigine” [16]. The likelihood function of the isotope pattern becomes:(4)

which measures the likelihood that a peptide with an theoretical isotope pattern *f*(*iso*) could have caused the observed value . The term  can be used as the isotope pattern matching score. However, it is critical to assign a reasonably good approximation of the noise variance. The noise variance generally scales with the peptide abundance with a power law . Various *p* have been reported in the literature [11,12]. The former suggested a poisson distribution on noise and thus *p* =1, the later suggested a quadratic form, *p* = 2, at high intensities for a Q-TOF MS. Note that these previous results are based on individual MS scans and are derived for specific instruments. When applying the peak picking algorithm, the selection of *p* should be adjusted. In the simulation section, we experimented with several possible values of *p* and found that *p* = 3 yields the best performance on the instrument that we experimented for low abundance proteins.

Note that in the past, the estimation of isotope pattern in algorithms such as [6] is based on local observations of the isotope pattern in one MS scan indexed by *t_r_* and at one charge state indexed by cs: *y_i_*(*t_r_, cs, iso*) = *A_i_ · C_i_*(*t_r_*) *· f*(*cs*) *· f*(*iso*) + *n* which has a smaller SNR. Not surprisingly, the performance is a lot worse than the result based on information combining algorithm we propose here which is shown in simulation results.

#### ICPD algorithm

The previous section introduced a method for estimating isotope pattern by combining all relevant information in different scans and charge states. Before the method can be applied, several preprocessing steps are necessary to complete the peak detection process. The ICPD algorithm is described as the following.

• Generate a peak candidate list *MassList.*

• For each entry in the *MassList,* extract their chromatographic peaks at different charge state and isotope positions.

• Estimate the isotope pattern by combining multiple scans and charge states, and calculate the isotope matching score.

• Produce an output candidate list annotated by various peak parameters such as isotope matching score.

• Apply a threshold on the matching score, and produce a list of detected peaks.

Now we explain each processing steps. 1. To generate a peak candidate list *MassList,* we first amass a list *LMZOverall* of all m/z values where a MS peak is centered. For high resolution data, this can be done by converting all MS scans to centroid data. Many software package provide this functionality and we utilized the mspeaks function provided by Matlab.

Then we group all m/z values that are within a bin of 2*dmz ppm* from each other. *dmz* is determined mostly by the mass accuracy of the instrument. Then, an elution time profile can be extracted for the m/z value within the bin. Then segments of the LC profile that contains at least *s* non-zero values are extracted. Here *s* is a lower bound for LC peak width. It is desirable to set it to a very small number (2 - 3) such that no peptides can be missed in the candidate list. Segments with a gap of *g* scans are combined. The tolerable gap in LC chromatographic gap depends on the instrument operating mode. If LC/MS and LC/MS/MS are collected together, ions may be directed to LC/MS/MS periodically which produces gaps in LC/MS. After segmentation and gap filtering in the LC profile process, the resulted segments are considered as peptide candidate peaks in the considered m/z value bin. The mean m/z value is recalculated within each segment. This process ensures that peptides with small mass difference within the bin that elute at different times are separated. Each peptide candidate is registered in a list called *MZlist.* Each entry of the *MZlist* contains the elution start time, end time, m/z value. Overlapping of bins of maximally *dmz ppm* is allowed. This could cause a peptide peak being registered twice in the *MZlist.* A subsequent merging step is performed if two peaks have m/z values that are within *dmz ppm* and the elution time period is overlapping.

Subsequently, this candidate *MZlist* is converted to the peptide candidate mass list *MassList* by assuming upto *CS* charge states. For example, if the maximum charge state considered is 4, then *MZlist* will be expanded four times by assuming each peptide candidate in *MZlist* has charge state 1, 2, 3 and 4. Thus, an entry in *MassList* contains a mass value of the peptide candidate as well as the elution time period.

2. To extract chromatographic peaks of a peptide candidate in the *MassList,* the m/z values at different isotope positions and different charge states are theoretically calculated first. For example, if the mass of the *i*th entry of *MassList* is *M_i_*, then its m/z value at charge state *cs* and isotope position *iso* can be calculated as *MZ*(*cs, iso, M_i_*) = (*M_i_* + cs * w_p_ + iso * w_n_)/*cs*. Then MS peak centroid falling within *±dmz* of the theoretically predicted values and within the elution time period of the peak candidate are extracted from the LC/MS datasets. These MS peaks are then sorted according to their elution time form the chromatographic peak of the peptide at the considered charge state and isotope position.

3. After extracting the chromatographic peaks, isotope pattern estimation can be performed as described in section .

4. An output list *OutList* is generated by annotating each candidate peptide in *MassList* with their isotope matching score as well as other parameters such as maximum peak intensity and peak volume.

5. User pick a threshold on the isotope matching score, and all entries that pass the threshold will be reported as detected peptide peaks.

## Competing interests

The authors declare that they have no competing interests.

## Authors contributions

Dr. Jianqiu Zhang developed and tested the ICPD algorithm, performed all computational simulations. Dr. William Haskins prepared the LC/MS UPS1 and UPS2 data sample used for testing, as well as provided Mascot searching results of UPS1 peptide identification information based on UPS1 LC/MS/MS data.
